# Comparison of outcomes following lobectomy, segmentectomy, and wedge resection based on pathological subtyping in patients with pN0 invasive lung adenocarcinoma ≤1 cm

**DOI:** 10.1002/cam4.4807

**Published:** 2022-05-15

**Authors:** Weijian Song, Yucheng Hou, Jianfeng Zhang, Qianjun Zhou

**Affiliations:** ^1^ Department of Thoracic Surgery, Shanghai Lung Cancer Center, Shanghai Chest Hospital Shanghai Jiaotong University, School of Medicine Shanghai China

**Keywords:** invasive lung adenocarcinoma, lobectomy, segmentectomy, wedge resection

## Abstract

**Purpose:**

We sought to analyze the prognostic significance of lung adenocarcinoma classification for patients with pathological N0 (pN0) lung invasive adenocarcinomas ≤1 cm who underwent surgical resection and investigate the optimal surgical procedure according to lung adenocarcinoma classification.

**Methods:**

A total of 1409 consecutive patients with resected pN0 invasive lung adenocarcinoma ≤1 cm were retrospectively reviewed. Comprehensive histologic subtyping was determined according to IASLC/ATS/ERS lung adenocarcinoma classification. Recurrence‐free survival (RFS) and overall survival (OS) were compared between patients receiving lobectomy, segmentectomy, and wedge resection.

**Results:**

RFS and OS favored lobectomy and segmentectomy compared with wedge resection in the entire cohort. Five‐year RFS rates were 100%, 98.2%, 97.3%, 77.8%, and 82.8% (*p* < 0.001) for lepidic, acinar, papillary, micropapillary, and solid predominant subtypes, while 5‐year OS rates were 100%, 98.4%, 98.1%, 88.9%, and 96.5% (*p* < 0.001), respectively. Multivariate analysis showed that adenocarcinoma predominant pathological subtype and CT appearance were independent prognostic factors for RFS, and surgical procedure was independent factor for both RFS and OS. Specifically, wedge resection showed worse survival compared with anatomical resection in patients with papillary, micropapillary, or solid predominant subtypes, whereas in patients with lepidic predominant and acinar predominant subtypes, wedge resection showed comparable RFS with anatomical resection.

**Conclusions:**

Anatomical resection showed better survival for patients with pN0 invasive lung adenocarcinoma ≤1 cm. For patients with invasive adenocarcinoma ≤1 cm in whom anatomical resection is not feasible, wedge resection could provide similar oncological effect when tumor is lepidic predominant or acinar predominant.

## Graphical abstract

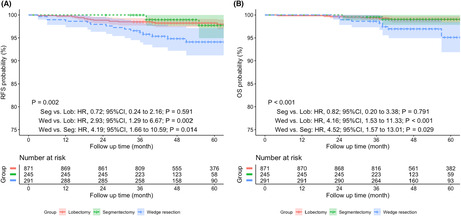

## 1 INTRODUCTION

Due to the wide application of high‐resolution CT (HRCT) scan, more small‐sized lung adenocarcinomas have been detected. The opportunity to detect invasive adenocarcinomas ≤1 cm has been increasing dramatically.^1^ Since 1995, lobectomy has been proved to be the standard surgical option for early‐stage (≤3 cm) non‐small cell lung cancer (NSCLC) rather than segmentectomy according to a randomized prospective clinical trial performed by the Lung Cancer Study Group.^2^ However, as the lung cancer spectrum shifts toward adenocarcinoma in decades, argument still exists about the optimal surgical procedure for small‐sized and early‐stage lung adenocarcinomas; whether sublobar resection or lobectomy should be applied for this subgroup has not yet been decided.

Recently, the 8th edition of the tumor, node, and metastasis (TNM) classification proposed by the International Association for the Study of Lung Cancer (IASLC),^3^ has further sub‐classified small‐sized tumor (previous T1a ≤ 2 cm) into T1a (≤1 cm) and T1b (≤2 cm) based on the significant survival difference. According to IASLC/America Thoracic Society (ATS)/ European Respiratory Society (ERS) classification, invasive adenocarcinoma is defined by the predominant histopathological subtypes. The prognostic importance of IASLC/ ATS/ ERS adenocarcinoma classification has been validated by several researches^4^, ^5^, ^6^; however, the optimal surgical treatment and oncological effect of each histopathological subtype in invasive lung adenocarcinoma ≤1 cm remain unclear because of the rarity.

Hence, in this retrospective study, we aimed to investigate the oncological outcomes of patients with invasive pN0 adenocarcinoma ≤1 cm undergoing lobectomy, segmentectomy, or wedge resection on the basis of the IASLC/ ATS/ ERS lung adenocarcinoma classification.

## 2 METHODS

### 2.1 Patients

**FIGURE 1:**
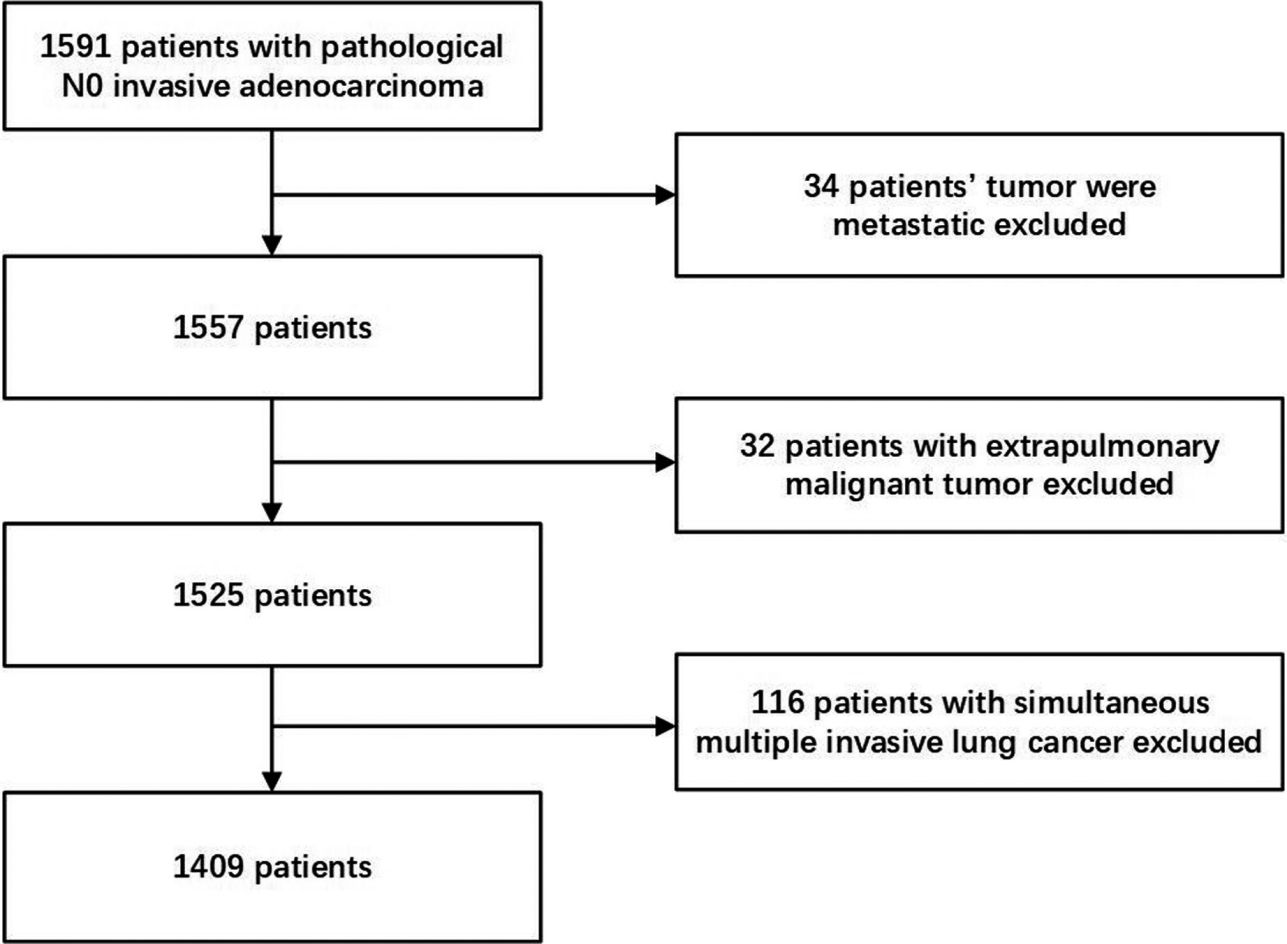
Selection and exclusion criteria of involved patients

We retrospectively evaluated 1409 patients who underwent surgical resection at the period of January 2013 to December 2017 and were diagnosed as pN0 invasive lung adenocarcinoma ≤1 cm in diameter on the final pathological report at Shanghai Chest Hospital, Shanghai Jiaotong University School of Medicine, Shanghai, China. Patients were excluded if the following conditions existed: 1. tumor size >1 cm on final pathological report; 2. metastatic tumors present; 3. combined with extrapulmonary malignant tumor; 4. multiple invasive lung cancer; 5. positive resection margin. (Figure 1). Ultimately, a total of 1409 patients with invasive lung adenocarcinoma no more than 1 cm were analyzed in this study. This study has been approved by the Clinical Research Ethics Committee of Shanghai Chest Hospital, Shanghai Jiaotong University School of Medicine, with the approval number: KS2011.

### 2.2 Preoperative and radiological evaluation

Chest CT scan, brain MRI, abdominal ultrasonography, and bone scintigraphy were performed for preoperative staging. Positron‐emission tomography (PET) scan was not routinely performed unless there was suspicious lymph node metastasis. For all the patients, findings of preoperative thin‐section CT (TSCT) were confirmed by two radiologists independently. Tumors were classified into pure glass‐ground opacity (pGGO), mixed glass‐ground opacity (mGGO), and pure‐solid tumor by the extent of GGO on TSCT. Lymph node no longer than 1 cm in the short axis on TSCT was defined as clinical N factor negative. Mean CT value was the average CT value of tumor on the layer with maximum diameter. All thin‐section CT image layers were 1 mm or 1.25 mm in thickness.^7^

### 2.3 Surgical procedure and resection margin

Lobectomy with systemic or lobe‐specific lymph node dissection was our primary procedure,^8^ and all patients were clinical N0 before operation (minor axis of lymph node ≤1 cm on thin‐section CT image). All the operations were done by minimally invasive approach. The decision of sublobar resection was made if CT appearance showed pure GGO or GGO dominant. Wedge resection was performed in occasion when patient was elderly or with compromised cardiopulmonary function. The sufficient resection margin of at least 1 cm was secured in sublobar resection which was recorded in the pathological reports. Margin recurrence was defined as tumor recurrence at the original surgical margin. The identification of the surgical margin is mainly by the metal shadow of the anastomotic nail on the CT image, and the identification of recurrence is confirmed by PET‐CT or pathological biopsy.

### 2.4 Histopathological evaluation

All pathological slices in the present study were reviewed and reclassified according to the TNM classification of malignant tumors, eighth edition by two experienced pathologists who were blinded to patients' clinical information. According to 2015 WHO classification of lung adenocarcinoma, invasive lung adenocarcinomas were divided into lepidic, acinar, papillary, micropapillary (MP), and solid (S) predominant subtypes based on the histological component pattern with the largest percentage.^9^ Visceral pleural invasion (VPI) was confirmed by Elastica van Gieson staining to assess the preservation of elastic layer of the visceral pleura.

### 2.5 Follow‐up after surgery

All the patients received postoperative follow‐up examination regularly including serum tumor marker, chest HRCT, and abdominal ultrasonography every 6 to 12 months. When recurrence or metastasis was suspected, further evaluation was performed, including CT or PET scan, bone scanning, and brain MRI. Local recurrence was defined as occurrence within the ipsilateral hemithorax including lung, lymph node, and pleura. Distant recurrence was defined as distant organ metastases.

In this study, the outcomes of interest included recurrence‐free survival (RFS) and overall survival (OS). RFS was the interval from surgery until recurrence of lung cancer and OS was the time from surgery until death no matter any reasons.

### 2.6 Statistical analysis

For continuous variables, two‐sample *t*‐test was used to compare the difference and ANOVA was used to compare the difference in variables of multiple groups. Pearson chi‐squared test was used for analyzing categorical variables. We used Kaplan–Meier method to analyze survival and recurrence. Log‐rank test was used to compare the survival curves between two groups. Cox proportional hazards model was applied to identify independent predictors. A *p* value of ≤0.1 in the univariate analysis was brought into multivariate model. A two‐sided *p* value of <0.05 was considered to have significant difference. The resulting statistical information of different subgroup variables was presented using forest plot. SPSS (version 25.0, IBMSPSS Inc., Armonk, NY) was used to conduct all statistical analyses and survival curves were drawn with R (version 3.5.2, R Foundation for Statistical Computing, Vienna, Austria).

## 3 RESULTS

### 3.1 Clinical and Histopathological findings

Table 1 shows the overall clinical and histopathological characteristics of patients with invasive lung adenocarcinoma ≤1 cm undergoing surgical resection according to predominant pathological subtype. Of the 1409 patients, there were 194 (13.8%) lepidic predominant adenocarcinomas, 792 (56.2%) acinar predominant adenocarcinomas, 385 (27.3%) papillary predominant adenocarcinomas, 9 (0.6%) micropapillary predominant, and 29 (2.1%) solid predominant. Two patients died within 10 days after surgery due to cardiovascular and cerebrovascular events, thus these two patients were excluded in survival analysis. Thirty‐eight (2.7%) patients were micropapillary/solid predominant adenocarcinomas. Micropapillary and solid predominant subtype were categorized together not only because of their similarity in prognosis (5‐year RFS rates: 77.8% vs. 82.8%, *p* = 0.588; 5‐year OS rates: 88.9% vs. 96.5%, *p* = 0.643) and pathological differentiation grade, but also the rarity in tumors less than 1 cm (nine cases and 29 cases, respectively in a total of 1409 patients).

**TABLE 1:** Demographics and clinical characteristics according to histopathological subtype of patients with invasive lung adenocarcinoma ≤1 cm

Characteristics	Lepidic (*n* = 194)	Acinar (*n* = 792)	Papillary (*n* = 385)	MP/S (*n* = 38)	*p*‐value
Age					
≤65	158 (81.4%)	651 (82.2%)	325 (84.4%)	33 (86.8%)	0.660
>65	36 (18.6%)	141 (17.8%)	60 (15.6%)	5 (13.2%)	
Sex					
Male	58 (29.9%)	241 (30.4%)	146 (37.9%)	19 (50%)	**0.006**
Female	136 (70.1%)	551 (69.6%)	239 (62.1%)	19 (50%)	
Smoking history					
Yes	36 (18.6%)	168 (21.2%)	95 (24.7%)	10 (26.3%)	0.302
No	158 (81.4%)	624 (78.8%)	290 (75.3%)	28 (73.7%)	
FEV1%Pred	92.16 (84.75–99.56)	92.03 (89.97–94.08)	91.41 (88.22–94.60)	83.40 (73.23–93.57)	0.474
Tumor location					
Upper and Middle	145 (74.7%)	525 (66.3%)	243 (63.1%)	24 (63.2%)	**0.044**
Lower	49 (25.3%)	267 (33.7%)	142 (36.9%)	14 (36.8%)	
ASA score					
I‐II	170 (87.6%)	714 (90.2%)	347 (90.1%)	32 (84.2%)	0.499
III‐IV	24 (12.4%)	78 (9.8%)	38 (9.9%)	6 (15.8%)	
Tumor size (mm)	9.40 (9.26–9.55)	8.72 (8.62–8.82)	8.97 (8.84–9.10)	8.46(7.95–8.97)	**<0.001**
CT appearance					
pGGO	40 (20.6%)	68 (8.6%)	40 (10.4%)	0 (0%)	**<0.001**
mGGO	151 (77.8%)	609 (76.9%)	295 (76.6%)	9 (23.7%)	
Solid	3 (1.5%)	116 (14.6%)	50 (13.0%)	29 (76.3%)	
Maximun CT value	−169 (−198 to −140)	−96 (−110 to −81)	−90 (−110 to −70)	35 (1–70)	**<0.001**
Mean CT value	−464 (−490 to −438)	−345 (−362 to −327)	−339 (−363 to −315)	−92 (−175 to 8)	**<0.001**
CEA	1.97(1.76–2.17)	1.78(1.66–1.91)	2.00(1.83–2.18)	1.84(0.99–2.68)	0.160
Surgical procedure					
Lobectomy	119 (61.3%)	477 (60.2%)	250 (64.9%)	27 (71.1%)	0.322
Segmentectomy	40 (20.6%)	135 (17.0%)	65 (16.9%)	5 (13.2%)	
Wedge resection	35 (18.0%)	180 (22.7%)	70 (18.2%)	6 (15.8%)	
VPI					
Present	0 (0%)	15 (1.9%)	8 (2.1%)	3 (7.9%)	**0.010**
Absent	194 (100%)	777 (98.1%)	377 (97.9%)	35 (92.1%)	
STAS					
Present	2 (1.0%)	6 (0.8%)	1 (0.2%)	0 (0%)	0.623
Absent	192 (99.0%)	786 (99.2%)	384 (99.7%)	38 (100%)	
Adjuvant chemotherapy					
Yes	0 (0%)	10 (1.3%)	3 (0.8%)	2 (5.3%)	**0.029**
No	194 (100%)	782 (98.7%)	382 (99.2%)	36 (94.7%)	

Abbreviations: ASA, American society of anesthesiologists; CEA, carcinoembryonic antigen; FEV1%Pred, forced expiratory volume in 1 second/predicted; mGGO, mixed Ground‐glass opacification; pGGO, pure Ground‐glass opacification; STAS, spread through air space; VPI, visceral pleural invasion.

Bold values represent significant statistical differences.

With regard to surgical procedure, the 5‐year RFS rates in the lobectomy, segmentectomy, and wedge resection groups were 98.0%, 97.7%, and 94.1%, respectively (*p* = 0.002), while the 5‐year OS rates in the lobectomy, segmentectomy, and wedge resection groups were 99.2%, 99.1%, and 95.1%, respectively (*p* < 0.001).

There were significant differences among the four groups with respect to sex (*p* = 0.006), tumor size (*p* < 0.001), CT appearance (*p* < 0.001), mean and maximum CT value (*p* < 0.001), and VPI (*p* = 0.010). Male patients were more likely to present with poor component, and tumor with micropapillary/solid predominant component showed smaller tumor size, more solid appearance and higher maximum/mean CT value on HRCT. Patients with MP/S predominant component were at high risk of VPI.

### 3.2 Prognosis

**FIGURE 2:**
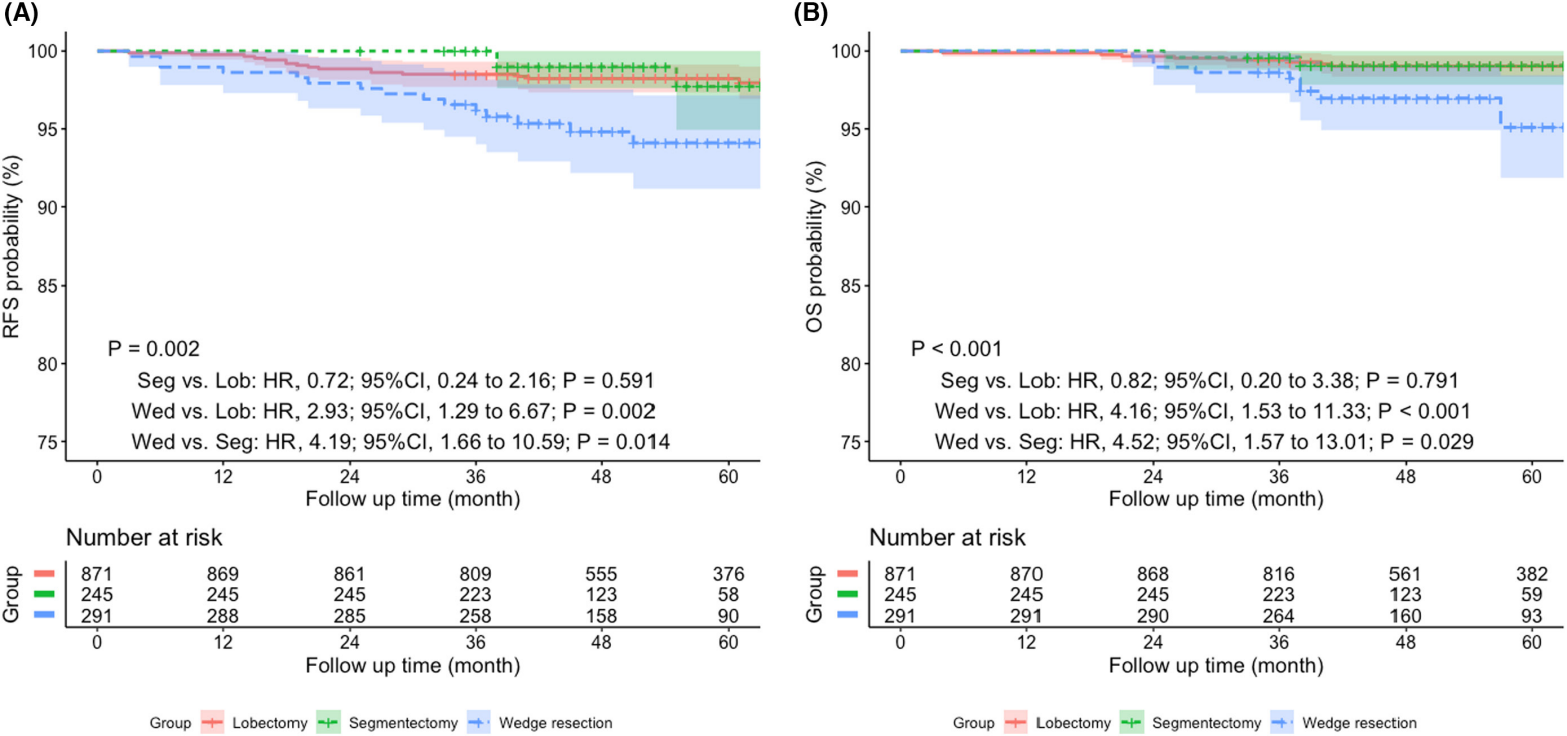
Prognostic impact on (A) RFS and (B) OS among patients undergoing lobectomy, segmentectomy, or wedge resection. HR, Hazard ratio; Lob, Lobectomy; OS, Overall survival; RFS, Recurrence‐free survival; Seg, Segmentectomy; Wed, Wedge resection

The median follow‐up time of the entire cohort was 50.9 months. The survival analysis by log‐rank test suggested that lobectomy and segmentectomy rather than wedge resection was significantly associated with better RFS (wedge resection vs. lobectomy: HR, 2.93; 95% CI, 1.29 to 6.67; *p* = 0.002; wedge resection vs. segmentectomy: HR, 4.19; 95% CI, 1.66 to 10.59; *p* = 0.014) and OS (wedge resection vs. lobectomy: HR, 4.16; 95% CI, 1.53 to 11.33; *p* < 0.001; wedge resection vs. segmentectomy: HR, 4.52; 95% CI, 1.57 to 13.01; *p* = 0.029), while no statistical difference was shown between those with segmentectomy and lobectomy (RFS: HR, 0.82; 95% CI, 0.20 to 3.38; *p* = 0.791; OS: HR, 0.72; 95% CI, 0.24 to 2.16; *p* = 0.591). (Figure 2).

Five‐year RFS rates were 100%, 98.2%, 97.3%, 77.8%, and 82.8% (*p* < 0.001) for lepidic, acinar, papillary, micropapillary, and solid predominant subtypes, while 5‐year OS rates were 100%, 98.4%, 98.1%, 88.9%, and 96.5% (*p* < 0.001) for each subtype, respectively.

Table 2 presents the results of Cox proportional hazard regression of RFS and OS of patients with pN0 lung invasive adenocarcinoma ≤1 cm. Both max CT value (*p* < 0.001) and mean CT (*p* < 0.001) value were strongly correlated with CT appearance, thus CT value was not included in the multivariate Cox regression analysis. Multivariate analysis showed sex (*p* = 0.032), CT appearance (*p* < 0.001), surgical procedure (*p* = 0.004), and predominant pathological subtype (*p* = 0.011) were independent prognostic factors for RFS. For OS, multivariate analysis showed that surgical procedure (*p* = 0.007) and CT appearance (*p* = 0.038) were independent prognostic factors.

**TABLE 2:** Cox regression model to predict OS and RFS of p‐N0 lung invasive adenocarcinoma ≤1 cm

Variables	Univariate		Multivariate	
	HR (95% CI)	*p*‐value	HR (95% CI)	*p*‐value
RFS				
Age (>65)	2.80 (1.38–5.65)	**0.004**	1.88 (0.89–3.97)	0.100
Sex (male)	2.96 (1.49–5.86)	**0.002**	2.14 (1.06–4.32)	**0.032**
Smoking	0.68 (0.32–1.41)	0.296		
ASA score (III‐IV)	0.26 (0.04–1.92)	0.188		
Tumor location (lower lobe)	1.03 (0.50–2.12)	0.933		
Tumor size	1.12 (0.86–1.46)	0.391		
CT appearance		**<0.001**		**<0.001**
Solid	1.00 (Reference)		1.00 (Reference)	
pGGO	0.14 (0.03–0.59)	**0.008**	0.20 (0.04–0.92)	**0.039**
mGGO	0.12 (0.06–0.24)	**<0.001**	0.20 (0.09–0.43)	**<0.001**
CEA	1.39 (0.75–2.59)	0.301		
Surgical procedure		**0.004**		**0.004**
Lobectomy	1.00 (Reference)		1.00 (Reference)	
Segmentectomy	0.71 (0.21–2.43)	0.582	0.86 (0.275–2.98)	0.806
Wedge resection	2.97 (1.47–6.02)	**0.002**	3.30 (1.55–7.04)	**0.002**
Path subtype		**<0.001**		**0.011**
MP/S	1.00 (Reference)		1.00 (Reference)	
Lepidic	0.05 (0.01–0.24)	**<0.001**	0.18 (0.03–0.99)	**0.049**
Acinar	0.11 (0.05–0.27)	**<0.001**	0.22 (0.09–0.58)	**0.002**
Papillary	0.08 (0.03–0.24)	**<0.001**	0.19 (0.06–0.58)	**0.004**
VPI	5.06 (1.55–16.58)	**0.007**	2.42 (0.72–8.14)	0.155
OS				
Age (>65)	3.13 (1.37–7.16)	**0.007**	2.09 (0.87–4.98)	0.098
Sex (male)	2.46 (1.10–5.49)	**0.028**	1.88 (0.81–4.32)	0.141
Smoking	1.10 (0.41–2.94)	0.854		
ASA score (III‐IV)	0.04 (0.01–14.92)	0.290		
Tumor location (lower lobe)	0.80 (0.35–1.83)	0.595		
Tumor size	0.95 (0.72–1.26)	0.714		
CT appearance		**0.001**		**0.038**
Solid	1.00 (Reference)		1.00 (Reference)	
pGGO	*		*	
mGGO	0.22 (0.10–0.48)	**<0.001**	0.32 (0.14–0.77)	**0.011**
CEA	1.17 (0.24–5.72)	0.843		
Surgical Procedure		**0.002**		**0.007**
Lobectomy	1.00 (Reference)		1.00 (Reference)	
Segmentectomy	0.88 (0.19–4.03)	0.868	0.95 (0.21–4.43)	0.951
Wedge resection	4.19 (1.80–9.73)	**0.001**	3.76 (1.57–9.01)	**0.003**
Path Subtype		0.292		0.770
MP/S	1.00 (Reference)		1.00 (Reference)	
Lepidic	**		**	
Acinar	0.36 (0.08–1.55)	0.168	0.74 (0.16–3.53)	0.709
Papillary	0.20 (0.04–1.04)	**0.055**	0.46 (0.08–2.60)	0.381
VPI	3.82 (0.89–16.34)	**0.071**	1.59 (0.35–7.29)	0.549

^a^ No patients with CT performed as pGGO died thus the Hazard ratio was incalculable.

^b^ No patients with lepidic predominant adenocarcinoma died thus the Hazard ratio was incalculable.

Abbreviations: ASA, American society of anesthesiologists; CEA, carcinoembryonic antigen; CI, confidence interval; HR, hazard ratio; MP/S, micropapillary or solid; OS, overall survival; RFS, recurrence‐free survival; VPI, visceral pleural invasion.

Bold values represent significant statistical differences.

**FIGURE 3:**
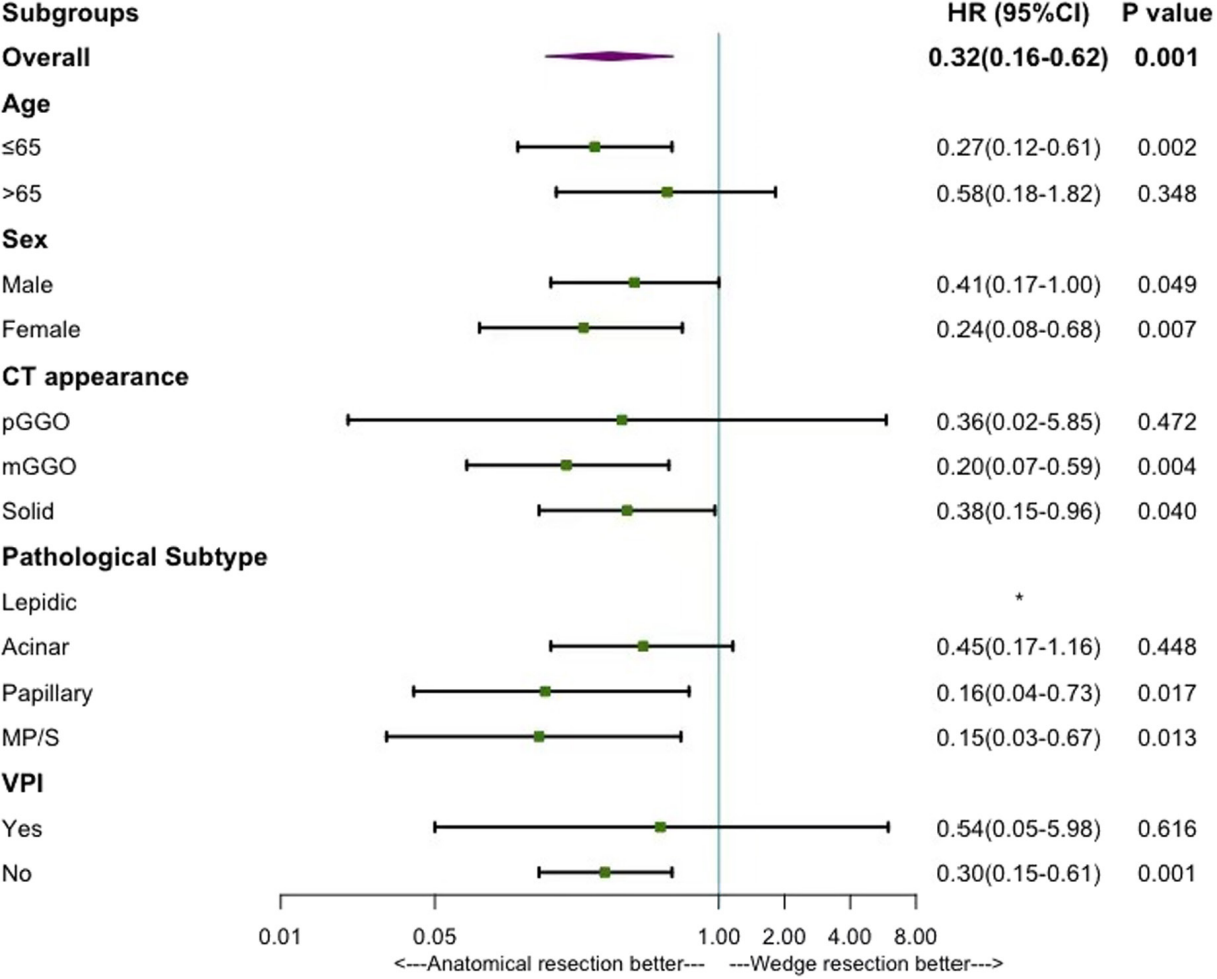
The effect of anatomical resection or wedge resection on RFS based on different subgroup variables. CI, confidence interval; HR, Hazard ratio; mGGO, mixed glass‐ground opacity; MP/S, micropapillary or Solid; pGGO, pure glass‐ground opacity; RFS, Recurrence‐free survival; VPI, visceral pleural invasion. *None of the patients who underwent wedge resection in lepidic group recurred so that the Hazard ratio was incalculable

**FIGURE 4:**
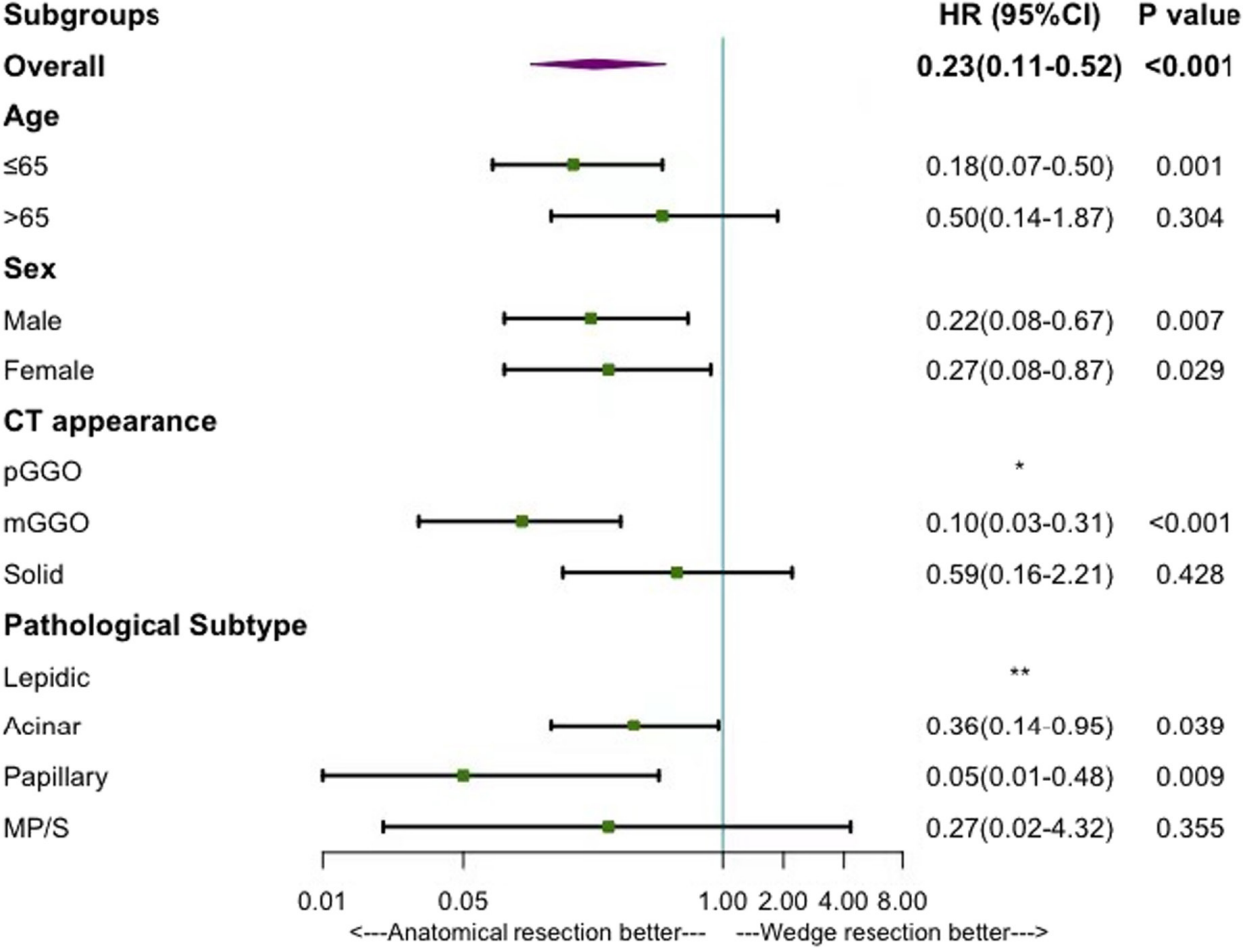
The effect of anatomical resection or wedge resection on OS based on different subgroup variables. CI, confidence interval; HR, Hazard ratio; mGGO, mixed glass‐ground opacity; MP/S, micropapillary or Solid; OS, Overall survival; pGGO, pure glass‐ground opacity. *: No patients with CT performed as pGGO died thus the Hazard ratio was incalculable. **: No patients with lepidic predominant adenocarcinoma died thus the Hazard ratio was incalculable

According to the results of survival analysis and Cox regression, segmentectomy had comparable prognosis to lobectomy, thus we combined lobectomy and segmentectomy into anatomical resection for further analysis. Multivariate Cox regression analysis demonstrated that variables, including age, sex, CT appearance, and pathological subtype were all independent prognostic factors of both RFS and OS, while additionally, VPI was an independent prognostic factor of RFS. To rule out the effects of these variables and further validate the effect of surgical procedure on RFS and OS, we conducted the subgroup analysis based on these variables. Significant RFS benefit was found in patients accepted anatomical resection who were with papillary and MP/S predominant pathological subtype (papillary: HR: 0.16; 95% CI: 0.04–0.73, *p* = 0.017; MP/S: HR: 0.15; 95% CI: 0.03–0.67, *p* = 0.013) rather than lepidic or acinar predominant pathological subtype (none of the patients who underwent wedge resection in lepidic group recurred so that the HR was incalculable; acinar: HR: 0.45; 95% CI: 0.17–1.16, *p* = 0.448). (Figure 3, Figure 4).

**FIGURE 5:**
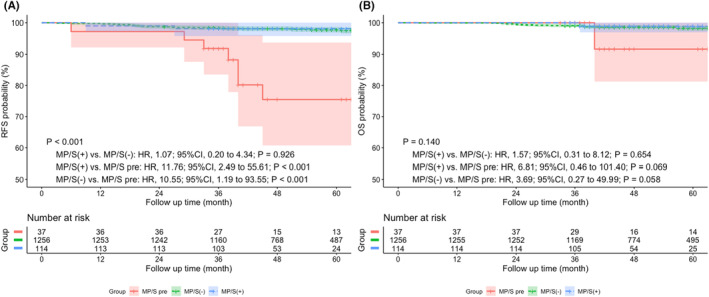
Prognostic impact on (A) RFS and (B) OS in MP/S predominant, MP/S positive (nondominant), and MP/S negative patients with invasive lung adenocarcinoma ≤1 cm. CI, confidence interval; HR, Hazard ratio; MP/S pre, micropapillary or solid predominant; MP/S(−), micropapillary or solid negative; MP/S(+), micropapillary or solid positive (nondominant); OS, Overall survival; RFS, Recurrence‐free survival

Considering that micropapillary or solid component as minor component was also considered as a risk factor for early lung adenocarcinoma, we classified all the patients into MP/S predominant group (MP/S pre), MP/S positive (not predominant) group (MP/S [+]), and MP/S negative group (MP/S [−]). There was no significant difference in survival between MP/S (−) and MP/S (+) groups (OS: HR, 1.83; 95% CI, 0.39 to 8.53; *p* = 0.546; RFS: HR, 1.18; 95% CI, 0.308 to 4.56; *p* = 0.819). While a significant worse RFS rate was shown in MP/S pre group compared with MP/S (+) group and MP/S (−) group. MP/S (+) versus MP/S pre: HR, 11.76; 95% CI, 2.49 to 55.6; *p* < 0.001; MP/S (−) versus MP/S pre: HR, 10.55; 95% CI, 1.19 to 93.55; *p* < 0.001) (Figure 5.)

As shown in Table 3, patterns of recurrence were listed in details according to the predominant pathological subtypes. For all the 536 patients underwent sublobar resection, no patients had a margin distance of less than 10 mm.

**TABLE 3:** Patterns of recurrence in patients undergoing sublobar resection with invasive lung adenocarcinoma ≤1 cm based on predominant pathological pattern

Characteristics	Lepidic (*n* = 194)		Acinar (*n* = 792)		Papillary (*n* = 385)		MP/S (*n* = 38)	
Surgical Procedure	Anatomical (*n* = 159)	Wedge (*n* = 35)	Anatomical (*n* = 612)	Wedge (*n* = 180)	Anatomical (*n* = 315)	Wedge (*n* = 70)	Anatomical (*n* = 32)	Wedge (*n* = 6)
Locoregional Recurrence	1 (0.6%)	0	7 (1.1%)	6 (3.3%)	2 (0.6%)	1 (1.4%)	1 (3.1%)	3 (50%)
Distant Metastasis	1 (0.6%)	0	4 (0.7%)	1 (0.6%)	1 (0.3%)	3 (4.3%)	2 (6.3%)	1 (16.7%)
No metastasis	157 (98.7%)	35 (100%)	601 (98.2%)	173 (96.1%)	312 (99.0%)	66 (94.3%)	29 (90.6%)	2 (33.3%)
*p* value	0.801		0.126		**0.010**		**0.001**	

Abbreviation: MP/S, micropapillary or solid.

Bold values represent significant statistical differences.

## 4 DISCUSSION

Although the number of small‐sized lung adenocarcinoma is expected to increase in this decade as the results of widely used CT screening, the optimal surgical approach of these early‐stage tumors remains unclear.^10^, ^11^, ^12^, ^13^, ^14^ Limited resection might be a definitive local treatment. The debate focuses on which kind of limited resection is appropriate for patients with certain clinicopathological features to balance the local recurrence and pulmonary function loss in current clinical scenario.^12^, ^15^, ^16^ Previous studies compared the oncologic efficiency between lobectomy and sublobar resection without considering the pathological subtyping, and more importantly, noninvasive cases were not excluded.^17^, ^18^, ^19^

IASLC/ATS/ERS histologic classification has been shown to be a prognostic factor for patients with adenocarcinoma.^9^, ^20^, ^21^ However, few studies had shed light on the prognostic value of IASLC/ATS/ERS classification for patients with invasive lung adenocarcinoma ≤1 cm treated with different extent of surgical resection. In this study, we demonstrated that the survival superiority favors anatomical resection (lobectomy and segmentectomy) compared with wedge resection for invasive lung adenocarcinoma ≤1 cm. The JCOG0802 study reported the prognosis of segmentectomy and lobectomy for lung tumors less than 2 cm with CTR >0.5, suggesting that the local recurrence rate of segmentectomy is higher than that of lobectomy. However, in the JCOG0802 study, the average tumor diameter was 1.6 cm, while this study focused on pulmonary nodules below 1 cm. Moreover, we revealed the difference in oncological results among different predominant pathologic subtypes on the basis of surgical approach for invasive adenocarcinoma ≤1 cm. Specifically, wedge resection showed worse survival compared with anatomical resection in patients with papillary predominant subtype and in patients with micropapillary or solid predominant subtypes, whereas in patients with lepidic predominant and acinar predominant subtypes, wedge resection showed comparable RFS and OS with anatomical resection. According to the results of JCOG0802, the recurrence rate of segmentectomy was significantly higher than that of lobectomy in tumors below 2 cm and with CTR >0.5, but there was no significant difference in OS. The reason may be similar to the conclusions in this study. Anatomic resection may be due to greater trauma and more death from non‐tumor factors despite a lower recurrence rate. The pathological subtype can affect the recurrence of the sub‐centimeters invasive lesions but has not yet affected OS in this study, which probably can be attributed to the comprehensive treatment after recurrence.

A pooled‐analysis performed by Zhang et al. showed the inferior survival for sublobar resection for NSCLC ≤1 cm.^22^ In a retrospective study using SEER database, Chen et al. proved the better survival for lobectomy compared with sublobar resection. For sublobar resection, similar survivals were observed between wedge resection and segmentectomy for NSCLC ≤1 cm, however, more than 1/3 patients were not adenocarcinomas.^23^ Hattori et al. did not prove the relationship between surgical approach and survival in a retrospective research containing 328 patients with sub‐centimeter NSCLC.^24^ The difference in survival for different pathological subtypes removed by sublobar resection might be one of the major reasons of these discrepancies. Of importance, surgical approach, pathological subtype, CT appearance remained independent predictors of recurrence in multivariate analysis in our research. Interestingly, tumor size was not a prognostic factor for both RFS and OS in our research. Our results suggested that optimal surgical procedure for lung adenocarcinoma might relate to its pathological subtype in early‐stage adenocarcinoma ≤1 cm.

Papillary, micropapillary, or solid predominant subtype adenocarcinoma have been proved to have worse prognosis compared with lepidic predominant subtype.^25^, ^26^ Both as the mid‐differentiation adenocarcinoma, we found the difference in prognosis between acinar and papillary predominant subtypes for wedge resection in invasive adenocarcinoma ≤1 cm. Previous reports have also showed the difference in survival with regard to surgical approach between acinar and papillary predominant subtypes in stage I patients.^25^, ^27^ Our results might be attributed to the sufficient margins or less lymph node evaluation requirement for the wide wedge resection when the patient is acinar predominant pathological patterns. In our study, 8% patients with lung adenocarcinoma ≤1 cm containing a micropapillary or solid component of 5% or more (but not predominant). For these patients, sublobar resection did not show an inferior survival. In fact, to report the low proportion of micropapillary or solid component during frozen section is not a regular procedure. Thus, our results suggested that patient with invasive adenocarcinoma ≤1 cm who underwent sublobar resection with enough margin may not need another extended resection if micropapillary or solid component was reported on final report.

Although micropapillary and solid histologic subtype were recognized as a factor of poor prognosis, the relationship between proportion of high‐grade component and prognostic impact in adenocarcinoma ≤1 cm were not well studied because of the scarcity of the patients with such tumors. In this study there were 38 (2.7%) micropapillary and solid predominant cases, and both predominant subtypes showed similar shorter RFS (*p* < 0.001), so that we put them together for survival analysis due to the scarcity. Lee et al. had reported that even a small component of micropapillary pattern will have a significant prognostic impact on OS.^28^ Chen et al. had showed wedge resection was associated with higher risk of recurrence compared with anatomic resection for sub‐centimeter lung adenocarcinoma with a micropapillary component of 5% or greater in a cohort 311 patients.^29^ Contrarily, unlike micropapillary and solid predominant subtypes, patients with adenocarcinoma ≤1 cm with micropapillary and/or solid component (not predominant) treated with wedge resection did not show inferior RFS and OS compared with anatomical resection (*p* = 0.58) in our study. It is noteworthy that the wedge resections in our cohort had a resection margin more than 1 cm, which might suggest the local infiltration for invasive adenocarcinoma ≤1 cm with micropapillary and solid component (not predominant) is less than 1 cm. In this study, we still use the cutoff value of micropapillary or solid component as 5% because of the previous standard for pathological diagnosis, although the newly modification of grading system to supplement the adenocarcinoma histological classification has raised the cutoff value of high‐grade patterns to 20%.^25^, ^30^

Findings on thin‐section CT were once regarded as preoperative indicator for the choice of surgical approach in small‐sized NSCLC. However, a pure GGO ≤1 cm sometimes was diagnosed as invasive adenocarcinoma, and vice versa 20% patients with a pure‐solid lesion ≤1 cm were proved to be noninvasive adenocarcinomas, indicating the discrepancy between the radiological and pathological findings.^31^ However, the presence of a GGO component on CT scan does not always indicate a good prognosis. Chen et al. also proved CT appearance was not an appropriate method to evaluate the invasiveness of early‐stage pulmonary nodule.^32^ CTR, the extent of consolidation on thin‐section CT, has been used to stratify malignant behavior and is a qualitative measurement via subjective judgment.^33^ However, for GGO tumor ≤1 cm, it is difficult to determine whether it contains solid components or the proportion of solid components due to its small size. A significant difference was shown in max CT value and average CT value among pathological predominant subtype groups (*p* < 0.001), and micropapillary and solid pathological subtype showed extraordinarily higher max and average CT value. Future studies are needed to explore the value of new technology such as Artificial Intelligence to predict malignant potential preoperatively.

To the best of our knowledge, this study is one of the largest retrospective study focusing on stage IA1 (≤1 cm) invasive lung adenocarcinoma and aiming to validate the prognostic impact of the IASLC/ATS/ERS histopathological subtype. This is a single institution study reviewing patients across a period of 8‐year which provides significant survival data to support our analysis. Our institution is experienced in lung surgery and performs over 20,000 lung surgeries annually. Nevertheless, we should acknowledge several limitations in this study. First, because of the noncontrolled, retrospective nature of our study, some biases were inevitable even though statistical methods were applied to adjust for the covariates. Second, as a single‐center study, validation from another independent institution is warranted. Third, the classification of this study was based on postoperative pathological report, in fact, reporting the presence of micropapillary or solid predominant component during intraoperative frozen diagnosis is not the regular procedure. Future study will be focused on determining the micropapillary or solid feature based on artificial intelligence, circulating tumor cell /DNA, and other technologies. Finally, the follow‐up period is relatively short, we will continue to follow‐up in future studies, differences in recurrence may further lead to differences in OS through prolonged follow‐up.

In conclusion, anatomical resection is recommended over wedge resection in invasive lung adenocarcinoma ≤1 cm. Wedge resection is suitable only if the lesion is lepidic predominant. If the patient is acinar predominant with compromised physical condition, wedge resection could be a choice with caution, otherwise, lobectomy or segmentectomy should be performed. Prospective studies focusing on the screening of lepidic and acinar predominant component by preoperative examination are necessary in the future.

## CONFLICT OF INTEREST

The authors declare no conflict of interest.

## AUTHOR CONTRIBUTIONS

Weijian Song: Conceptualization, Methodology, Writing‐Original Draft; Yucheng Hou: Software, Formal analysis, Visualization; Jianfeng Zhang: Data Curation, Validation; Qianjun Zhou: Writing‐ Review & Editing.

## ETHICS STATEMENT

This study has been approved by the Clinical Research Ethics Committee of Shanghai Chest Hospital, Shanghai Jiaotong University School of Medicine, with the approval number: KS2011.

## Data Availability

The data that support the findings of this study are available from the corresponding author upon reasonable request.
